# B-cell lymphoma gene regulatory networks: biological consistency among inference methods

**DOI:** 10.3389/fgene.2013.00281

**Published:** 2013-12-16

**Authors:** Ricardo de Matos Simoes, Matthias Dehmer, Frank Emmert-Streib

**Affiliations:** ^1^Computational Biology and Machine Learning Laboratory, Faculty of Medicine, Health and Life Sciences, Center for Cancer Research and Cell Biology, School of Medicine, Dentistry and Biomedical Sciences, Queen's University BelfastBelfast, UK; ^2^Institute for Bioinformatics and Translational Research, UMITHall in Tirol, Austria

**Keywords:** gene regulatory network, C3Net, BC3Net, Aracne, GPEA, statistical inference

## Abstract

Despite the development of numerous gene regulatory network (GRN) inference methods in the last years, their application, usage and the biological significance of the resulting GRN remains unclear for our general understanding of large-scale gene expression data in routine practice. In our study, we conduct a structural and a functional analysis of B-cell lymphoma GRNs that were inferred using 3 mutual information-based GRN inference methods: C3Net, BC3Net and Aracne. From a comparative analysis on the global level, we find that the inferred B-cell lymphoma GRNs show major differences. However, on the edge-level and the functional-level—that are more important for our biological understanding—the B-cell lymphoma GRNs were highly similar among each other. Also, the ranks of the degree centrality values and major hub genes in the inferred networks are highly conserved as well. Interestingly, the major hub genes of all GRNs are associated with the G-protein-coupled receptor pathway, cell-cell signaling and cell cycle. This implies that hub genes of the GRNs can be highly consistently inferred with C3Net, BC3Net, and Aracne, representing prominent targets for signaling pathways. Finally, we describe the functional and structural relationship between C3Net, BC3Net and Aracne gene regulatory networks. Our study shows that these GRNs that are inferred from large-scale gene expression data are promising for the identification of novel candidate interactions and pathways that play a key role in the underlying mechanisms driving cancer hallmarks. Overall, our comparative analysis reveals that these GRNs inferred with considerably different inference methods contain large amounts of consistent, method independent, biological information.

## 1. Introduction

To date, a vast amount of gene regulatory network (GRN) inference methods are being developed with the future goal to establish qualitative and quantitative procedures for a structural, biological and experimental validation of the inferred networks (Friedman, 2004; Wille et al., 2004; Werhli et al., 2006; Margolin and Califano, 2007; Yip et al., 2010; Zhang et al., 2011; Emmert-Streib et al., 2012). One of the most conservative approaches for GRN inference was introduced with the C3Net (Altay and Emmert-Streib, 2010, 2011) method that inferres at most one interaction (edge) for each gene with the strongest mutual dependency. An extension of C3Net was introduced by the bagging (Breiman, 1996; Zhang and Singer, 2010) approach BC3Net (de Matos Simoes and Emmert-Streib, 2012) that allows to aggregate ensembles of C3Net networks that are inferred from bootstrap (Efron and Tibshirani, 1994; Davison and Hinkley, 1997) datasets. The main advantage of a C3Net and BC3Net over many other methods is the intuitive interpretation of the inferred interactions that correspond to gene-pairs with the strongest significant mutual dependency, present in the data. Notably, a C3Net GRN has the property to infer very sparse, modular networks with a preference for interactions in the periphery of the network corresponding to genes with a less complex mutual dependency structure.

In de Matos Simoes and Emmert-Streib (2011), C3Net was used to infer GRNs from simulated gene expression data using a known underlying network structure. This study demonstrated that interactions (edges) of genes with a low number of direct neighbors (low degree) are more likely to be inferred correctly compared to interactions of genes with a large number of direct neighbors. From this observation one can presume that the interaction periphery of the unknown gene network is more prominently represented in an inferred GRN due to the lower complexities of the gene expression dependencies between the genes. However, the underlying gene network is unknown when a GRN is inferred from real biological gene expression data. Thus, the periphery and the center of the gene network is restricted to known experimental interactions that provide only a static and incomplete representation of the gene network. Furthermore, in de Matos Simoes et al. (2012) it was shown that the giant connected component (GCC) of the GRN using C3Net is highly enriched with membrane associated proteins. This observation suggested that the periphery of a gene network represents, to some extend, also the physical periphery of the biological cell that is centered around signaling receptors that represent the major hubs of the GRN.

**Network Inference**

- Q: What types of biological networks have been inferred in the paper?
- A: We use gene expression data from B-cell lymphoma and infer GRNs.
- Q: How was the quality/utility of the inferred networks assessed?
- A: We compare the inferred GRNs with a protein-protein interaction network and a transcriptional regulatory network. Furthermore, we compare 3 GRNs among each other to identify their similarity. This analysis is conducted by using the Gene Ontology database and a variety of additional databases.
- Q: How were these networks validated?
- A: All networks are analyzed computationally and statistical hypotheses testing is employed to test various hypotheses about the network structure and the biological function of the investigated GRNs.

When comparing different methods with each other for inferring GRN it is important to conduct this comparison on similar grounds. For this reason, we are comparing in this paper only methods with each other that employ statistical hypothesis testing (Lehman, 2005; Young and Smith, 2005) and utilizing mutual information to estimate the interactions within regulatory networks. In this way we are avoiding a potential bias that could result from comparisons between networks with a different meaning.

The performance of GRN inference methods have been often compared using simulated data from biological or simulated network structures (Van den Bulcke et al., 2006; Schaffter et al., 2011; Emmert-Streib, 2013). One major problem with a simulation-based analysis is that the assumed mechanisms to simulate gene expression are only partially understood biologically, leaving a certain uncertainty about the resulting properties of the expression data. On the other hand, when real data are used, the underlying network structure remains unknown or highly incomplete. Furthermore, differences between inferred GRN using different methods may be negligible due to small sample sizes of real data sets and the presence of noise in these gene expression data.

Of great importance is the question “what” and “how consistent” is the information that can be extracted from a given large-scale gene expression data set to generate novel data-driven hypotheses. Unfortunately, to date, frequently, targets for wetlab studies are chosen based on the popularity of key genes rather than on the information within data sets. However, a non-data driven hypothesis ignores the limitations of an underlying data set to resolve known and unknown gene relationships. Furthermore, the efforts that have been performed for the validation of GRNs where mostly focusing on individual interactions, such as transcription factor target gene interactions (e.g., for MYC). To our knowledge, the most prominent genes appearing in a GRN, e.g., the actual hub genes, have not been considered for experimental validation.

In our study, we infer a C3Net, BC3Net and Aracne B-cell lymphoma GRN from a large-scale gene expression data set (Basso et al., 2005). We provide a structural and a functional comparison between the sparse, modular network structure inferred by C3Net and the more densely connected BC3Net and Aracne GRNs. Furthermore, we discuss the role of the hub genes and known cancer genes, such as MYC, we find in the inferred GRNs.

The paper is organized as follows. In the next section, we discuss the data we use for our analysis, the network inference methods and statistical measures we use for our analysis. In the results section, we present a comparative analysis and discuss differences between the 3 inferred GRNs and 2 reference networks (a PPN and a TRN). This article finishes with a discussion.

## 2. Materials and methods

### 2.1. Gene expression data

For our study, we use the gene expression data with the GEO (Barrett et al., 2011) accession GSE2350 from Basso et al. (2005). The data set includes transformed and untransformed B-cell lymphoma samples. For our analysis, we consider only samples for which raw gene expression data in form of Affymetrix CEL files are available. From the total of 387 samples of the GSE2350 dataset, 344 samples were available in a CEL file format from the hgu95a and hgu95av2 chip platform. The data were preprocessed as described in detail in de Matos Simoes and Emmert-Streib (2011). Probeset identifiers were mapped to entrez gene symbols when available using the *org.Hs.eg.db* R-package (Carlson, 2013). Multiple probesets that mapped to the same gene were summarized using their median value. The final gene expression data set comprises 9684 genes and 344 samples. We subsequently applied a copula transformation to the processed gene expression data, as described in Margolin et al. (2006).

### 2.2. Inference of gene regulatory networks

For the inference of the B cell lymphoma GRN, we use 3 mutual information-based GRN inference methods: C3Net, BC3Net and Aracne (Margolin et al., 2006; Altay and Emmert-Streib, 2010; de Matos Simoes and Emmert-Streib, 2012). Mutual information (MI) for all gene pairs is computed using a Pearson estimator (Meyer et al., 2007; Olsen et al., 2009),

(1) $$I\left(X,Y\right)=-\frac{1}{2}\log\text{​}\left(1-\rho^{2}\right),$$

where ρ is the Pearson correlation coefficient.

#### 2.2.1. Null-distribution of mutual information values

In order to determine the statistical significance of the mutual information values between genes we test for each pair of genes the following null hypothesis.

*H*^*I*^_0_: The mutual information between gene *i* and *j* is zero.

Because we are using a nonparametric test we need to obtain the corresponding null distribution for *H*^*I*^_0_ from a randomization of the data. Principally, there are several ways to perform such a randomization. Here we permute the sample and gene labels for all genes of the entire expression matrix at once. In de Matos Simoes and Emmert-Streib (2011) we investigated three different randomization schemes and found that the randomization procedure applied here [in de Matos Simoes and Emmert-Streib (2011) called RM3] leads to similar results as other procedures that are computationally more demanding.

#### 2.2.2. C3Net

**Figure 1:**
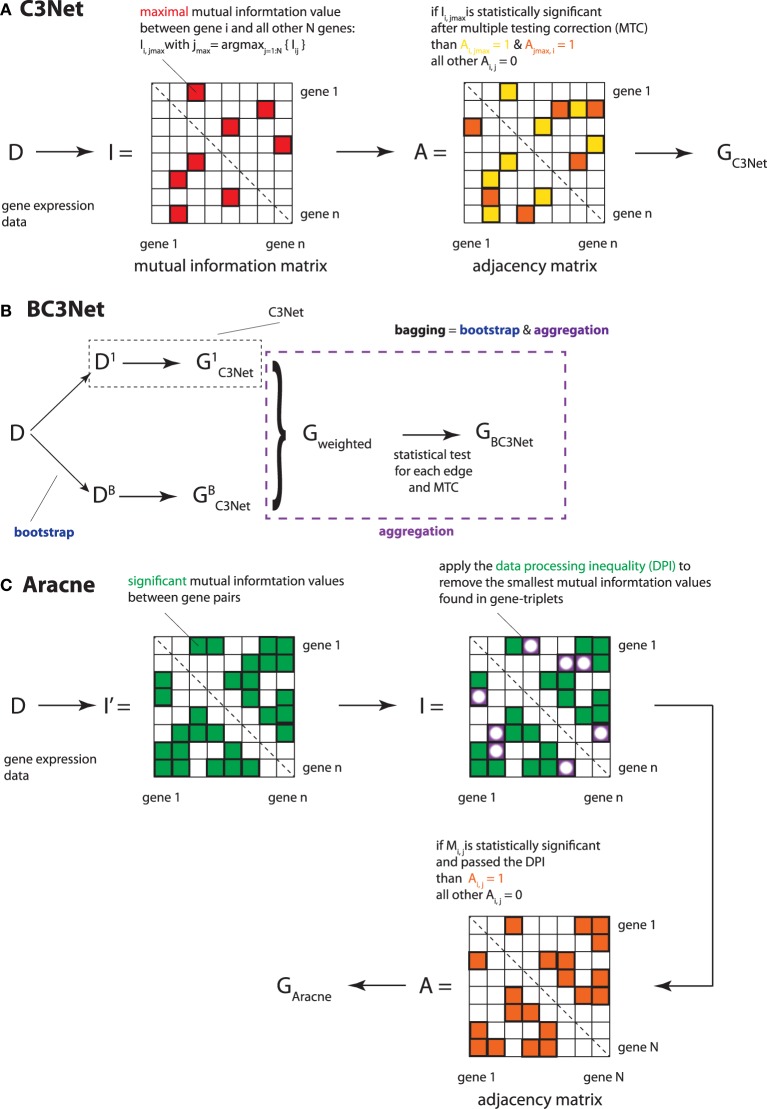
**Overview of the 3 applied inference methods and their key methodological analysis steps. (A)** C3Net, **(B)** BC3Net and **(C)** Aracne.

The C3Net (Conservative Causal Core) algorithm consists of three main steps (Altay and Emmert-Streib, 2010, 2011). In the first step, mutual information values among all gene pairs are estimated. For this, we use a Pearson estimator for mutual information values, as given in Equation 1. In the second step, we select for each gene only the largest mutual information interaction (see Figure 1, indicated by the red elements in matrix *I*). This interaction corresponds also to the most significant gene among the neighbor edges. Third, we apply a non-parametric significance test for the mutual information values of the largest elements. The null distribution for this test is obtained from a randomization of the sample labels in the gene expression matrix. We use a significance level of α = 0.05 in combination with a Bonferroni multiple testing correction (Dudoit and van der Laan, 2007).

Since C3Net employs mutual information values as test statistics among genes, there is no directional information that can be inferred thereof. Hence, the resulting network *G*_C3Net_ is undirected and unweighted (corresponding to a symmetric, binary adjacency matrix *A*; as indicated by the orange and yellow elements in Figure 1). For a detailed explanation of C3Net and its technical details, the reader is referred to Altay and Emmert-Streib (2010, 2011).

#### 2.2.3. BC3Net

The BC3Net (de Matos Simoes and Emmert-Streib, 2012) algorithm is a bagging (Breiman, 1996) version of C3Net (Altay and Emmert-Streib, 2010, 2011). Briefly, BC3Net consists of 2 major steps. In the first step, a bootstrap ensemble of B data sets is generated. For each data set in the ensemble a GRN is inferred using C3Net; see Figure 1. In step two, the resulting ensemble of networks is combined into a weighted network, where the weights in this network *G*_weighted_ describe the ensemble consensus rate for an edge in the bootstrap ensemble. Then, we apply a binomial test to all edges in the weighted network and retain only edges that are statistically significance for a significance level of α = 0.05 that pass a Bonferroni multiple testing correction (see Figure 1B—aggregation). This results into the final network *G*_BC3Net_. For a statistically detailed description, the reader is referred to de Matos Simoes and Emmert-Streib (2012).

#### 2.2.4. Aracne

The Aracne (algorithm for the reconstruction of accurate cellular networks) algorithm (Basso et al., 2005; Margolin et al., 2006) consists of two main steps. In step one, it estimates the mutual information values between all gene pairs and identifies their statistical significance. In Figure 1, these elements are represented as green elements in the matrix *I*′. In step two, all gene-triples (*ijk*), i.e., three genes with significant mutual information values, are used in combination with the *data processing inequality* (DPI) (Cover and Thomas, 1991) for thinning the resulting network. Specifically, for each triplet (*ijk*), the edge corresponding to the lowest mutual information value *I*_1_ = *I_i′j′_*, with (*i′j′*) = argmin {*I_ij_*, *I_jk_*, *I_ik_*}, is eliminated from the mutual information matrix *I* (in Figure 1 indicated by the white circles) and the adjacency matrix *A*, if it is smaller than the second smallest mutual information value *I*_2_, adjusted by a factor (1−ϵ), i.e.,

(2) $$A_{i^{\prime }j^{\prime }}=A_{j^{\prime }i^{\prime }}=\{\begin{array}{c} 0I_{i^{\prime }j^{\prime }}\leq I_{2}\left(1-\epsilon \right)\\ 1\text{ otherwise.} \end{array}$$

Here 0 ≤ ϵ ≤ 1. The introduction of this step has been motivated by the so called *data processing inequality* (DPI) (Cover and Thomas, 1991). The DPI is a relation between mutual information values, which means loosely that a post-processing of data cannot increase its information content. Specifically, one can show (Cover and Thomas, 1991) that the DPI for the following relation between the three random variables,

(3) X→Y→Z,

implies that *I*(*X*, *Z*) ≤ *I*(*X*, *Y*). Due to the fact that the criteria in Equation 2 is for ϵ > 0 less stringent than the DPI (Equation 3), ϵ is called *tolerance parameter*.

In order to ensure an unique solution that is independent of the order of the selected gene-triples, the procedure starts by listing all possible gene-triplets that can be found from the significant mutual information values after step one. Then, all of these gene-triplets are tested sequentially. Hence, the results of these tests have no influence on subsequent tests and the formation of gene-triplets.

For our practical application of Aracne, we use the standalone java executable Aracne2 (Basso et al., 2005; Margolin et al., 2006) available from (http://wiki.c2b2.columbia.edu/califanolab/index.php/Software/Aracne) to infer a GRN. For Aracne, we use the recommended parameter settings for this data set, listed in the following: For the mutual information estimator a kernel width of *w* = 0.12918 is defined with *b* = 6 bins. The significance threshold for MI was *t* = 0.064394 with a *p*-value threshold of *p* = 1.0*e* − 7. Aracne considers the removal of indirect interactions between a triplet of genes by applying the *data processing inequality* (DPI) with a tolerance parameter that is set to ϵ = 0.15.

### 2.3. Experimental interactions: reference networks

We use a meta collection of protein-protein interactions provided by iRefIndex (Razick et al., 2008). iRefIndex gathers protein interactions from BIND, BioGrid, DIP, HPRD, IntAct, MINT, MPact, MPPI and OPHID. Uniprot and refseq Ids were converted to entrez gene symbols using the *org.Hs.eg.db* R package (Carlson, 2013). If an identifier could not be mapped directly to entrez identifiers, the HUGO gene symbol was used. The remaining identifiers that could not be directly mapped to entrez gene symbols were not used. The resulting undirected protein network we use for our analysis includes a total of 185, 433 protein-protein interactions for 15,233 proteins.

Furthermore, we use a transcriptional regulatory network (TRN) provided by the HTRidb database comprising a collection of experimentally validated transcription factor target gene interactions (Bovolenta et al., 2012). The database comprises a total of 51,871 interactions for 284 transcription factors, regulating 18,302 genes.

In the results section, we use these two experimental networks as *reference networks* to compare them with the inferred GRNs.

### 2.4. Network centrality measures

In the following, we describe 4 network-based measures we use for our analysis, namely, (A) degree centrality, (B) edge density, (C) transitivity and (D) assortativity. For a more detailed description see (Newman, 2010; Emmert-Streib and Dehmer, 2011).

The (A) degree centrality is defined as the total number of direct neighbors of a vertex *v_i_* (gene). Formally, the degree centrality of *v_i_* in an undirected network is given by Newman (2010),

(4) $$C_{1}(v_{i})=\sum_{j=1}^{n}A_{ij},$$

where the adjacency matrix of the network is given by *A* and *n* is the total number of genes. That means *C*_1_(*v_i_*) of node *v_i_* is just the number of connections that node *v_i_* has to other nodes in the network. Frequently, this is briefly called the *degree* of a node.

The (B) edge density of a network is the number of edges divided by the maximal number of possible edges. For an undirected network, this number of possible edges is given by *n*(*n* − 1)/2, whereas *n* is the total number of genes. Hence, the edge density is a global measure for the connectivity of a network, whereas small values indicate sparsely connected networks and high values indicate densely connected networks.

The (C) transitivity centrality value of a vertex *v_i_*, also called the local clustering coefficient, measures the proportion of edges of the direct neighbors of *v_i_* in a clique of *k* vertices. The local clustering coefficient is given by Watts and Strogatz (1998),

(5) $$C_{3}(v_{i})=\frac{2|\{e_{ij}\}|}{k(k-1)},$$

where |{*e_ij_*}| is the number of edges for vertex *v_i_* to all its direct neighbors *v_j_*, and $\frac{k(k-1)}{2}$ corresponds to the total number of edges in a clique of *k* vertices. Formally, the transitivity of a vertex is that probability that two neighbors of this vertex are connected with each other. Informally, this can be translated in “friends of mine are friends too,” if a “friend” is defined as “connected with.”

Finally, the (D) assortativity measure is the Pearson correlation coefficient of the degree centrality between the connected vertices in a network (Newman, 2002),

(6) $$r=\frac{M^{-1}\sum_{i}j_{i}k_{i}-{\left[M^{-1}\sum_{i}\frac{1}{2}(j_{i}+k_{i})\right]}^{2}}{M^{-1}\sum_{i}\frac{1}{2}\left(j_{i}^{2}+k_{i}^{2}\right)-{\left[M^{-1}\sum_{i}\frac{1}{2}\left(j_{i}+k_{i}\right)\right]}^{2}}.$$

Here *j_i_* and *k_i_* correspond to the degrees of the vertices at the end of edge *i*, and *M* is the total number of edges in the network. We would like to remark that Equation 6 is symmetric in *j_i_* and *k_i_*. Informally, the assortativity is a global measure that gives positive values when—in average—vertices connect to other vertices that have a *similar* degree (e.g., high to high and low to low), and it has negative values—in average—when vertices connect to other vertices that have a *dissimilar* degree.

### 2.5. Degree centrality pathway analysis

We define the test statistic δ, as the average degree centrality in the GRN, for a set of *k* genes defined by a Gene Ontology term. For an undirected network, δ is given by,

(7) $$\delta_{obs}=\frac{1}{k}\sum_{i=1}^{k}(\sum_{j=1}^{n}A_{ij}),$$

where *A* is again the adjacency matrix of the network.

For each gene set (resulting from a GO term), the null distribution of δ is obtained from randomizations of the gene labels in the GRN. The *p*-value is estimated from the fraction of randomizations with a larger value than the test statistic, δ_obs_, for a given term in the GRN, i.e.,

(8) $$p=P\left(\delta \geq \delta_{obs}\right).$$

For each GO term, *R* = 10,000 randomizations are performed. We set the *p*-value to *p* = 0.0001 = 1/*R* in cases when none of the randomizations exceed the test statistic for a given term. We perform a multiple hypothesis correction using the FDR by Benjamini and Hochberg (1995).

### 2.6. Drugbank

For the major hub genes in a GRN, we tabulated the associated drugs from the drugbank database (Knox et al., 2011). We use the drugbank version from july 2013. The drug to target protein links were extracted from *all_target_ids_all.csv* and the drugnames from *drug_links.csv*. We map uniprot identifiers to entrez gene symbols using org.Hs.eg.db R-package (Carlson, 2013).

## 3. Results

### 3.1. Global properties of gene regulatory networks

For the B-cell lymphoma gene expression data set in Basso et al. (2005), we infer 3 GRN using C3Net, BC3Net and Aracne. For the 3 inferred networks, we estimated the edge-density, maximal node degree, size of the giant component (GCC), assortativity and transitivity (Table 1A). Here, the GCC is the largest subnetwork and its size corresponds to the number of genes in this subnetwork.

**Table 1:** **(A) Global network properties of the B-cell lymphoma C3Net, BC3Net and Aracne GRN. **(B)** Edge-overlap between the 3 GRN**.

	**C3Net**	**BC3Net**	**Aracne**
**(A)**			
Number of genes	9684	9684	9684
Number of edges	9221	57, 905	320, 668
Edge-density	1.9×10^−5^	1.2 × 10^−3^	6.8 × 10^−3^
Max degree	46	169	2198
Number of components	463	8	1
Size of GCC	884	9668	9684
Assortativity	−0.144	−0.0195	0.0543
Transitivity	0.089	0.000	0.230
**(B)**			
C3Net	9221 (100%)	9215 (99.93%)	9167 (99.41%)
BC3Net	9215 (15.91%)	57, 905 (100%)	52, 777 (91.11%)
Aracne	9167 (2.86%)	52, 777 (16.46%)	320, 668 (100%)

*(A, B) For this table, we compare the number of edges in both networks divided by the total number of edges of the network in the row*.

As one can see from Table 1A, for these global measures the networks differ considerably for all measures. Specifically, the C3Net GRN has the lowest edge density (1.9 ×10^−5^) and it is composed of 463 separated network components (subnetworks). In contrast, the Aracne GRN has the highest edge density (6.8 × 10^−3^) followed by the BC3Net GRN (1.2 × 10^−3^). The assortativity coefficient shows a weak negative correlation for C3Net indicating a tendency that, e.g., genes with a high degree have a tendency to be connected with genes with a low degree. For BC3Net and Aracne this cannot be observed.

From a pairwise comparison of the 3 GRNs in Table 1B, we find that the C3Net GRN is a subnetwork of BC3Net and Aracne, with almost all edges (99%) represented in both networks (see Table 1B). Also the BC3Net and the Aracne GRN show a large overlap with over 91.11% (52,777/57,905) of common edges that are present in BC3Net. In contrast, only 16.46% (52,777/320,668) of the common edges are present in the Aracne GRN.

On a general note, we would like to add that the differing number of edges in the 3 inferred GRN is related to the different inference methods applied (see Methods section). Whereas C3Net aims only to infer the interactions within a GRN that are strongest, as emphasized by its name (Conservative Causal Core = C3), BC3Net is a bagged (Breiman, 1996) version of C3Net that is capable of exploiting also less strong signals by estimating their variability from an ensemble approach. Finally, Aracne employs an entirely different inference strategy than C3Net or BC3Net. Whereas C3Net aims only to infer the strongest interactions and BC3Net aims to *add* additional interactions by bagging C3Net, Aracne uses the *data processing inequality* to thinning all significant mutual information values. Hence, C3Net is the most conservative approach, Aracne is the most anti-conservative approach and BC3Net is situated in-between them.

The results in Table 1 indicate clearly that the 3 GRNs are considerably different among each other, if compared with global measures.

### 3.2. Functional analysis of B-cell lymphoma networks

Next, we investigate the functional similarity of the 3 GRNs. In order to identify the most prominently represented biological processes in the 3 B-cell lymphoma GRN, we perform a *gene pair enrichment analysis* (GPEA). The GPEA analysis tests the null hypothesis whether the number of interactions in a GRN connecting genes from the same GO term is similar to the number of interactions connecting genes from different GO terms. This is tested by a hypergeometric test.

We perform the GPEA using gene sets, defined by the Gene Ontology database, for the categories biological process (BP), molecular function (MF) and cellular component (CC). In addition, we use terms defined in the reactome database. Furthermore, we compare the results obtained for the C3Net, BC3Net and Aracne gene regulatory networks among each other.

In Table 2A, we show an overview of the number of significant terms identified using the GPEA. For example for GO BP, we find 124 significant terms for C3Net, 166 significant for BC3Net and 386 significant terms for Aracne. The number of significant terms is similar between C3Net and BC3Net. For the Aracne GRN, the number of significant terms is almost twice as large. The number of significant terms for the reactome is similar for all three networks comprising a total of 30% of the terms. For MF the number of significant terms is the lowest for the three networks comprising only 5% of the terms. Table 2B shows the overlap of significant terms for BP, MF, CC and reactome between C3Net, BC3Net, and Aracne. For all pairwise comparisons, we observe an overlap of >90% of significant terms between pairs of GRNs, except for MF.

**Table 2:** **(A) Functional enrichment using a GPEA for the C3Net, BC3Net, and Aracne GRN. Shown are the numbers of significant terms/number of total terms, and the percentage of significant terms. **(B)** Overlap percentage (%) of significant terms in the GPEA between the C3Net, BC3Net and Aracne gene regulatory networks**.

	**C3Net%**	**BC3Net%**	**Aracne%**
**(A)**			
BP	124/1673 (7.4)	166/2604 (6.3)	386/3565 (10.8)
CC	30/241 (12.4)	49/357 (13.7)	110/477 (23.1)
MF	8/308 (2.6)	25/535 (4.7)	38/774 (4.9)
Reactome	92/270 (34.1)	129/387 (33.3)	186/492 (37.8)
**(B)**			
C3Net vs. BC3Net	92.74	96.67	87.50	96.74
C3Net vs. Aracne	92.74	100.00	87.50	96.74
BC3Net vs. Aracne	97.59	97.96	64.00	99.22

**Figure 2:**
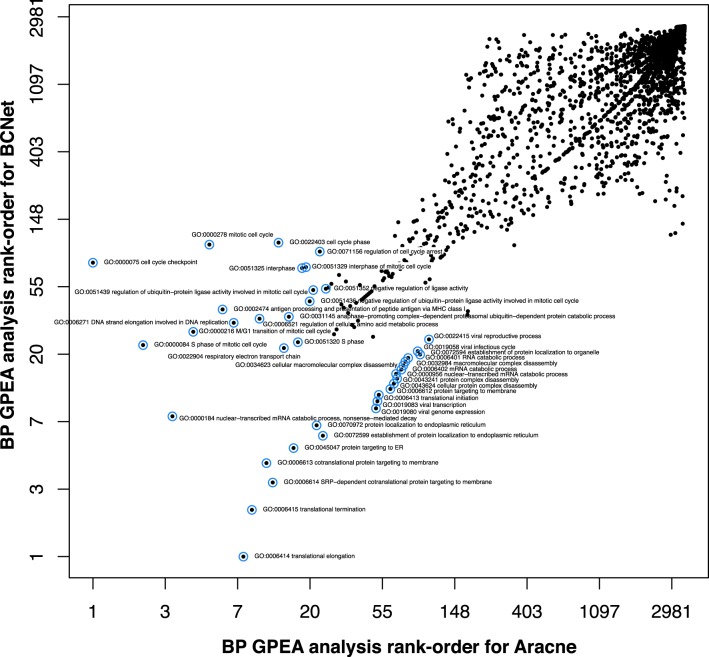
**Comparison of the rank-order of significant biological process (BP) GO terms from the GPEA analysis for BC3Net (*y*-axis) and Aracne (*x*-axis)**. The axis are log-transformed for a better visualization. The blue circles correspond to the top GO terms for Aracne and BC3Net.

Another interesting observation we make is that the rank-order of significant GO terms is highly correlated between C3Net and BC3Net (*r* = 0.88, *p* ≤ 2.2 × 10 − 16), but also the other two pairs of GRNs. For instance, Figure 2 shows a pairwise comparison of the rank-order of the GPEA analysis for BP terms between BC3Net and Aracne, whereas the topmost 25 pairs are highlighted in blue. That means for each network, we rank-ordered the analyzed GO terms according to their resulting *p*-values and we used these ranks as x-coordinates (Aracne) and y-coordinates (BC3Net) in Figure 2. On a technical note, we want to remark that we used logarithmically transformed (log-transformed) values to obtain a better visualization of the shown GO terms. However, because a logarithm is a monotonous function, the original rank-order of the GO terms remains unchanged by this transformation.

Biologically, from the top 25 BP GO terms in Figure 2 we observe a variety of significant biological processes for protein translation, targeting and protein complex disassembly, viral transcription and cell cycle. Interestingly, in contrast to the results from the global analysis of GRNs, the functional analysis indicates that all 3 GRNs are biologically quite similar to each other.

### 3.3. Experimental interactions: comparison with reference networks

In this section, we compare the 3 inferred GRNs with experimental networks (serving as reference networks). Specifically, we use a protein-protein interaction network (PPN) and a transcriptional regulatory network (TRN) for this comparison. The transcriptional regulatory network is obtained from the HTRIdb database of experimental validated transcription factor target gene interactions (Bovolenta et al., 2012). The database comprises a total of 284 transcription factors and 18,302 target genes comprising a total of 51,871 interactions. The PPN is from iRefIndex containing a total of 185, 433 protein-protein interactions among 15, 233 proteins; see the Methods section for more details.

An overview of the pairwise comparisons between the B-cell lymphoma GRNs and the TRN is shown in Table 3A and the comparison with the PPN is shown in Table 3B. The percentage of shared interactions for all 3 GRNs is very low, and ranges around 0.1%. However, only for C3Net the number of shared interactions with the TRN is significant. For the comparison between the PPN and the inferred GRNs the number of shared interactions is significant for all three GRNs and the percentage of shared interactions is in the range between 1% to 2%. Again for C3Net we observe the highest overlap of edges between the GRN and the TRN.

**Table 3:** **Network comparison between the 3 B-cell lymphoma GRNs and the (A) TRN and (B) PPN**.

**(A) Transcriptional regulatory network (TRN)**					
	**Shared genes (%)**	**Edges GRN**	**Edges TRN**	**Shared edges%**	***p*-value**
C3Net	7915	6361	22,668	8 (0.125)	0.045
BC3Net	7915	39, 507	22, 668	33 (0.084)	0.176
Aracne	7915	210, 036	22, 668	134 (0.064)	0.923
**(B) Protein protein network (PPN)**					
	**Shared genes (%)**	**Edges GRN**	**Edges PPN**	**Shared edges%**	***p*-value**
C3Net	7944	6429	100,074	145 (2.226)	0
BC3Net	7944	40,049	100,074	563 (1.406)	0
Aracne	7944	213,841	100,074	2110 (0.987)	0

*Here, shared genes and shared edges correspond to the genes and edges that can be found in a GRN and the (A) TRN and (B) PPN*.

#### 3.3.1. Correlation between the degree centrality of the GRNs and the reference networks

**Figure 3:**
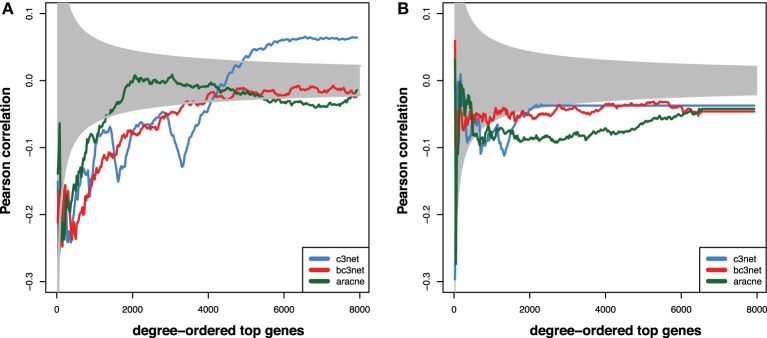
**Shown are Pearson correlation coefficients that are obtained for genes that are rank-ordered according the size of their degree centrality values in the GRNs compared to: (A) PPN and (B) TRN**. The ordering is from high to low degree centrality values and the size of the underlying profile vectors increases with the number of the gene rank. The gray area indicates correlation values that are not statistically significant for α = 0.05.

In this section, we study the correlation between the degree centrality value of genes that we find in the GRNs and the experimental reference networks, i.e., the TRN and the PPN, using the Pearson correlation coefficient. Specifically, we start with the topmost 25 genes in these networks and then increase the number of the genes sequentially in step sizes of 25 genes, until all genes are included. This corresponds to an averaging window with one fixed side and one sliding side that increases in steps of 25 genes to lower ranked genes. The results of this analysis are shown in Figure 3. In this figure, the gray area indicates correlation values that would *not* be statistically significant for a significance level of α = 0.05. In other words, all correlation values that are outside the gray area, are statistically significant. We obtained the values of the significance boundaries from using an assymtotic relation between a t-statistic, *t*, and a Pearson correlation coefficient, *r*, given by Sheskin (2004)

(9) $$r=\frac{t}{\sqrt{df+t^{2}}}.$$

Here *df* = *n* − 2 is the degree of freedom of the data for a profile vector of length *n*. It is important to note that also a t-statistic is a function of the degree of freedom, i.e., *t*(*df*). From selecting a significance level α one obtains for each profile vector of a certain length, *n*, the corresponding t-statistics, which gives via Equation 9 the corresponding values for the Pearson correlation coefficients. For our analysis we assumed a two-sided hypothesis explaining the symmetric values of the correlation around zero. As one can see from Figure 3, due to increasing sizes of the profile vectors for which correlations are assessed, these decision boundaries are not constant but are becoming narrower around zero when more genes are used in the analysis.

For all three GRNs and the PPN, we observe a tendency for the high-degree genes to show a statistically significant negative correlation to the degrees observed in the PPN (~−0.2, Figure 3A). For larger window sizes, the correlation slowly decreases for C3Net and BC3Net, but much faster for Aracne. Interestingly, C3Net assumes positive statistically significant correlation values (~0.05) for very large window sizes. The observations for the comparison between the three GRNs and the TRN are similar, however, less strong (see Figure 3B). In this case, all three GRN inference methods C3Net, BC3Net and Aracne retain their negative statistically significant correlation values, even for very large window sizes.

### 3.4. Hub genes

In this section, we study the major hub genes in the B-cell lymphoma GRNs inferred from C3Net, BC3Net and Aracne. Furthermore, we conduct a functional analysis to elucidate the role of the involved biological processes of the major hub genes.

**Figure 4:**
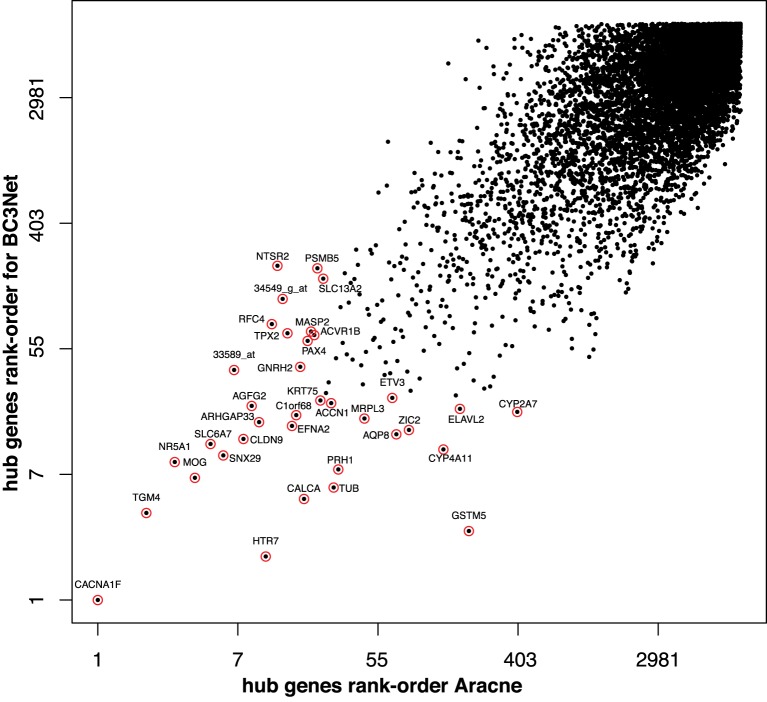
**Comparison of the rank-order of hub-genes for BC3Net (y-axis) and Aracne (x-axis)**. The axis are log-transformed for a better visualization. The red circles correspond to the top hub genes for Aracne and BC3Net.

We start by performing a global comparison of the degree centrality values for all genes between the C3Net, BC3Net and Aracne GRN. The largest global rank-order Spearman correlation coefficient for all genes is observed between C3Net and BC3Net (*r* = 0.72, *p* < 2.2 × 10^−16^) and for BC3Net and Aracne (*r* = 0.74, *p* < 2.2 × 10^−16^). The lowest correlation is observed between C3Net and Aracne (*r* = 0.54, *p* < 2.2 × 10^−16^). In Figure 4, we show the pairwise comparison between the rank-order of the degree centrality of all genes for BC3Net and Aracne. Interestingly, we observe no substantial difference between the degree rank of genes with the highest node degree. This holds for all pairwise comparisons between the three GRNs. That means all 3 GRNs contain essentially the same hub genes.

In general, the connection between hub genes for different physiological contexts, as represented by a protein network (PPI) or a GRN, is not well studied. In a PPI network, lethal proteins have been observed to have the tendency to form hubs (Jeong et al., 2001), whereas non-lethal disease associated proteins, which are putative drug targets, are more likely to reside at the periphery of a PPI network (Goh et al., 2007). Interestingly, in contrast to a PPI network, the GRN hub genes of the B-cell lymphoma GRNs have the tendency to be associated with signaling receptors, such as from the G-protein coupled receptor pathway that comprises promising drug targets in cancer (Lappano and Maggiolini, 2011).

We would like to note that the hub genes of the B-cell lymphoma GRNs, see Figure 4, are not restricted to signaling receptors and can also include a variety of transcription factors such as, e.g., ZIC2 and ELAVL2 (HuB). Although, the literature does not show studies investigating these genes specifically for B-cell lymphoma, several studies point to their importance for the development of tumors. For instance, ZIC2 was observed with a higher expression in malignant ovarian tumors (Marchini et al., 2012) and overexpression analysis showed oncogenic properties of ZIC2 to drive tumor growth in ovarian cancer (Marchini et al., 2012). Also, proteins of the ELAV gene family (Hu genes) such as ELAVL2 are tumor antigens that are investigated for early stage lung cancer detection (D'Alessandro et al., 2010). Hu genes are usually expressed in neuron cells and were found to have an ectopic expression in neurodendocrine tumors (Gultekin et al., 2000). However, the association between tumor progression and Hu gene expression remains unclear on the molecular level.

For the functional analysis of the GRN hub genes, we applied a non-parametric test to identify biological processes that are related to genes with a large degree centrality value in the GRN. We perform a permutation-based test that defines the average degree centrality from the GRN as test statistic for the gene set of a given GO term; see Equation 7. As a result, Table 4 shows the most significant biological process terms with the highest average degree centrality (δ_obs_) in the GRN (with FDR≤0.1). We observe a large variety of signaling related processes such as adenylate cyclase-modulating G-protein coupled receptor signaling pathway, cell-cell signaling, sensory perception, cell cycle processes (S phase), blood pressure regulation and macrophage differentiation.

**Table 4:** **Results for the degree centrality pathway analysis test for the BC3Net GRN**.

**GO**	**Term**	**δ_obs_**	**δ (avg)**	**Size**	***p*-value**	**FDR**
GO:0007188	Adenylate cyclase-modulating G-protein coupled receptor signaling pathway	17.5	11.95	86	0.0001	0.02877
GO:0007267	Cell-cell signaling	13.73	11.96	776	0.0001	0.02877
GO:0007600	Sensory perception	14.69	11.95	263	0.0001	0.02877
GO:0009581	Detection of external stimulus	21.06	11.95	52	0.0001	0.02877
GO:0009582	Detection of abiotic stimulus	21.77	11.96	47	0.0001	0.02877
GO:0009583	Detection of light stimulus	25.27	11.99	26	0.0001	0.02877
GO:0050877	Neurological system process	13.45	11.96	772	0.0001	0.02877
GO:0051320	S phase	16	11.95	119	0.0001	0.02877
GO:0007187	G-protein coupled receptor signaling pathway, coupled to cyclic nucleotide second messenger	16.6	11.96	111	0.0001	0.02877
GO:0007601	Visual perception	16.24	11.97	132	0.0001	0.02877
GO:0008217	Regulation of blood pressure	15.92	11.97	110	0.0001	0.02877
GO:0050906	Detection of stimulus involved in sensory perception	23.46	11.98	26	0.0001	0.02877
GO:0050953	Sensory perception of light stimulus	16.24	11.96	132	0.0001	0.02877
GO:0003073	Regulation of systemic arterial blood pressure	18.6	11.96	50	0.0002	0.04675
GO:0051606	Detection of stimulus	17.45	11.95	84	0.0002	0.04675
GO:0000216	M/G1 transition of mitotic cell cycle	17.85	11.95	65	0.0002	0.04675
GO:0007189	Adenylate cyclase-activating G-protein coupled receptor signaling pathway	19.51	11.96	41	0.0003	0.06233
GO:0000084	S phase of mitotic cell cycle	15.96	11.96	113	0.0003	0.06233
GO:0045649	Regulation of macrophage differentiation	32	11.92	10	0.0004	0.07874

We further studied whether major hub genes of a GRN are drugable by known drugs that are related to the treatment of B-cell lymphoma. For the 30 genes with the largest degree centrality in the B-cell lymphoma BC3Net we extracted associated drugs from the drugbank database (Knox et al., 2011). A variety of drugs were associated with 8 genes comprising calcium-channel blockers (calcium channel subunit CACNA1F), dopamine antagonists (serotonin receptor HTR7), metabolic compounds such as glutathione, NADH, L-proline and pituitary hormone analogues, see Table 5 for an overview.

**Table 5:** **Drug targets for major hub genes in the BC3Net B-cell lymphoma gene regulatory network, see Figure 4**.

**Target gene**	**Drugbank**	**Drugname**
CACNA1F	DB00393	Nimodipine
	DB00568	Cinnarizine
	DB00661	Verapamil
	DB01388	Mibefradil
	DB04855	Dronedarone
	DB04920	Clevidipine
HTR7	DB00216	Eletriptan
	DB00246	Ziprasidone
	DB00247	Methysergide
	DB00248	Cabergoline
	DB00334	Olanzapine
	DB00363	Clozapine
	DB00751	Epinastine
	DB01200	Bromocriptine
	DB01224	Quetiapine
	DB01238	Aripiprazole
	DB04946	Iloperidone
	DB06216	Asenapine
	DB06288	Amisulpride
	DB08815	Lurasidone
GSTM5	DB00143	Glutathione
TUB	DB02028	DB02028
NR5A1	DB04683	DB04683
	DB04752	Phosphatidyl ethanol
CYP4A11	DB00157	NADH
SLC6A7	DB00172	L-Proline
AVPR1B	DB00035	Desmopressin
	DB02638	Terlipressin

#### 3.4.1. MYC

**Figure 5:**
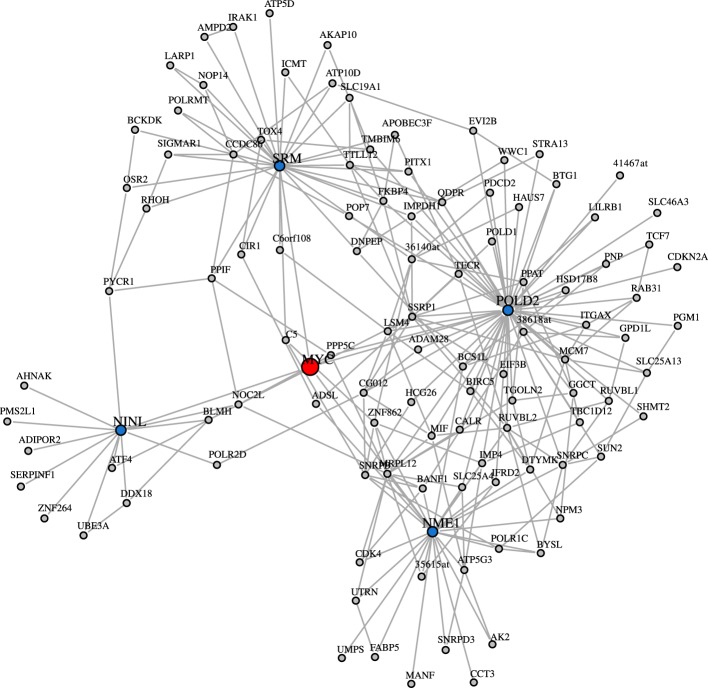
**BC3Net subnetwork including MYC (red) and its 2nd level nearest neighbors**. The first degree MYC neighbors are shown in blue and the 2nd degree MYC neighbors are shown in gray.

The study of Basso et al. (2005) provided a validation for some interactions of the transcription factor MYC. However, when considering the degree centrality values of MYC in the inferred networks, MYC has a low rank-order. Interestingly, this is consistent for all three inferred GRNs and holds also for the ranking of other network-based measures. In Table 6, the rank of MYC is shown for C3Net, BC3Net and Aracne (in decreasing order of the absolute degree value) for the degree centrality, betweenness and local transitivity. For example, MYC ranks for the degree centrality of the GRN for C3Net 3110 (9684), BC3Net 9322 (9684) and Aracne 2317 (9684). Here, the number in brackets corresponds to the total number of genes. In the C3Net GRN, we find that MYC has only one single direct neighbor. In the BC3Net GRN, MYC has 4 direct neighbors, namely, POLD2 (52 neighbors), NME1 (26 neighbors), SRM (30 neighbors), NINL (13 neighbors) (Figure 5). For the Aracne GRN, MYC has 68 direct neighbors. The direct neighbors of C3Net and BC3Net are also present in the Aracne GRN.

**Table 6:** **MYC rank (in decreasing order) for degree centrality, betweeness and transitivity for the C3Net, BC3Net, and Aracne GRN**.

**GRN**	**Degree rank**	**Betweeness rank**	**Transitivity rank**	
C3Net	3110 (1)	6955 (0)	1936 (0)
BC3Net	9322 (4)	2184 (4142.263)	3968 (0)
Aracne	2317 (68)	3397 (7778)	712 (0.23)

*The absolute values of the centrality measure are shown in parenthesis. In total, the maximal rank order is 9684 corresponding to the total number of genes*.

## 4. Discussion

In this paper, we conducted a structural and a functional analysis of B-cell lymphoma GRNs that were inferred using 3 mutual information-based inference methods, namely, C3Net (Altay and Emmert-Streib, 2010), BC3Net (de Matos Simoes and Emmert-Streib, 2012) and Aracne (Basso et al., 2005). On the global-level, our analysis revealed that the inferred B-cell lymphoma GRNs have major differences in their edge density, maximal degree, transitivity and assortativity. However, on the edge-level, the 3 GRNs were highly similar among each other, whereas the C3Net GRN and the BC3Net GRN represent almost a subnetwork of the Aracne GRN. The global differences in the edge densities can be mainly explained by the different inference strategies employed by the three methods (see Methods section) resulting always in GRNs with the following ordering in the number of edges; # edges_C3Net_ < # edges_BC3Net_ < # edges_Aracne_.

A C3Net GRN represents the *core* network structure of a GRN that considers only the strongest signal from a data set (Altay and Emmert-Streib, 2010). Although C3Net limits the analysis to a very sparse GRN structure, it provides a less complex and more clearly arranged structural organization of large GRN networks (de Matos Simoes et al., 2012). Furthermore, because only the strongest gene neighbors are considered for each gene, using C3Net or BC3Net, the number of putative indirect associations is highly reduced.

In our study, we compared also the biological functions that are significantly represented in GRNs. We observed high similarities between the GRN of C3Net, BC3Net and Aracne, where significant biological processes, cellular components and Reactome terms were overlapping with >90%. The tendency for Aracne to observe a larger number of significant terms for the GPEA analysis can be explained due to the larger edge-density that is beneficial for a GPEA analysis. Among the significant terms common in all 3 GRNs, we find biological processes for protein translation, targeting and protein complex disassembly, viral transcription and cell cycle.

Next, we compared the 3 inferred GRNs with 2 experimental networks (we called reference networks). Specifically, we compared the 3 GRNs with a protein-protein interaction network (PPN) and a transcriptional regulatory network (TRN). From this comparison, we determined the quantitative edge-overlap between the 2 reference networks and the 3 B-cell lymphoma GRNs. For the TRN, we observed ~ 0.1% of shared interactions with the GRNs. However, for the PPN, we observed a higher relative percentage of 1−2% of shared interactions. A reason for this low, but significant (see *p*-values in Table 3), overlap is three-fold. First, the used reference networks are not *condition specific* for B-cell lymphoma. For instance, many interactions in the PPN are obtained from *yeast-two-hybrid* (Y2H) experiments providing only information about the potential binding of proteins outside a particular cellular context (Maslov and Sneppen, 2002). Similarly, the experimentally verified interactions in the TRN provided by the HTRidb database are identified from a wide range of different normal (not pathological) physiological conditions. Second, a GRN provides only an average representation of the interactions across the spatial and temporal separation of the cellular processes that are reflected by the observed gene expression dependencies. Third, due to the different data types used to assemble a PPN (e.g., Y2H), TRN (e.g., ChIP-chip) and a GRN (gene expression) they are all different from each other. The relation between these networks has been studied systematically for the model organisms *S. cerevisiae* and *E.coli* in de Matos Simoes et al. (2013).

We compared the degree centrality of the GRNs to the PPN and TRN. For the PPN and the TRN, we observed a statistically significant negative correlation for the genes with the largest degree centrality, independent of the GRN inference method. That means, the major hub genes of a GRN have a tendency to relate to proteins with a low(er) degree in the PPN or TRN. This analysis suggest that proteins with few direct neighbor interactions have a stronger relationship in gene expression data for the corresponding genes that are connected in a GRN, which may more likely represent the periphery of the gene network. However, one major limitation of defining the degree centrality from a PPN network is that protein interactions are not well defined and gathered from multiple experimental methods for different interaction types that are not distinguished and largely incomplete.

In a PPI network lethal proteins have been observed to have a tendency to form hubs (Jeong et al., 2001), whereas non-lethal disease associated proteins, which are putative drug targets, are more likely to reside at the periphery of a PPI network (Goh et al., 2007). Our functional analysis to identify pathways with a significantly larger average degree centrality revealed pathways involved in the G-protein coupled receptor signaling pathway, sensory perception, cell-cell signaling and cell cycle. G-protein coupled receptors are prominent drug targets for a large catalogue of conditions such as cardiovascular related and neuropsychiatric disorders (Esposito et al., 2002; Albizu et al., 2010) and promising drug targets in cancer (Lappano and Maggiolini, 2011).

For example, the major hub gene CACNA1F, see Table 5, can be inhibited by a variety of channel blockers like nifedipine, amlodipine, verapamil, and diltiazem (Striessnig et al., 2010). Due to the importance of ion channels in signaling the calcium channel blockers are also being investigated for the treatment of B-cell lymphoma (Shamash et al., 1998). For CACNA1F 6 calcium channel blocking drugs were identified from drugbank. The combination of verapamil and antineoplastic agents is suggested to induce chemosensitivity in chemoresistant cells (Simpson, 1985). Furthermore, mibefradil was shown to slow tumor growth in glioblastoma cell lines (Keir et al., 2013). The serotonin receptor HTR7 is a G-protein coupled receptor. The drugs associated to HTR7 are dopamine antagonists used for neuropsychiatric disorders. GSTM5 is associated to Glutathione that is highly abundant and important for protecting the cell against free radicals, but also promote chemoresistance (Balendiran et al., 2004). A number of studies investigated the depletion of Glutathione following chemotherapy for increasing chemosensitization of cancer cells (Balendiran et al., 2004). Lastly, AVPR1B is associated to desmopressin which may impair metastasis of cancer cells (Gomez et al., 2006).

The hub genes of the B-cell lymphoma GRN are not restricted to signaling receptors and can also include transcription factors such as ZIC2 or RNA-binding proteins such as ELAVL2 (HuB). Although, the literature does not show studies investigating these genes specifically for B-cell lymphoma several studies point to their importance in tumorgenic processes. ZIC2 was observed with higher expression in malignant ovarian tumors (Marchini et al., 2012). Overexpression analysis showed oncogenic properties of ZIC2 to drive tumor growth in ovarian cancer (Marchini et al., 2012). Proteins of the ELAV gene family (Hu genes) such as ELAVL2 are tumor antigens that are investigated for early stage lung cancer detection (D'Alessandro et al., 2010). Hu genes are usually expressed in neuron cells and were found to have an ectopic expression in neurodendocrine tumors (Gultekin et al., 2000). However, the association between tumor progression and Hu gene expression remains unclear on the molecular level.

This discussion shows a potential application of the resulting GRNs. That means, major inferred hub genes could be used for the experimental validation of drugs to effect important biological pathways of B-cell lymphoma. In this way, data-driven hypothesis about drug targets could be derived from the inferred GRN (Ildirim et al., 2007; Hopkins, 2008; Ghosh and Basu, 2012). Additionally, in a similar way, hallmark pathways could be studied, because since the seminal work by Hanahan and Weinberg (2000, 2011) it is generally accepted that the molecular causes of cancer need to be approaches on this level, rather than on the level of individual genes.

Overall, our analysis sheds light on the biological similarity of GRNs inferred with C3Net, BC3Net and Aracne, and indicates that these network inference methods contain consistent biological information. This is a very important result, because it demonstrates the biological robustness of the information that can be reliably derived from such different GRNs, despite existing differences among various other aspects of such networks.

### 4.1. Data sharing

We provide the gene expression data, the inferred GRNs and the reference experimental networks from our analysis in the R-package BClymphomaGRN, available from CRAN.

### Conflict of interest statement

The authors declare that the research was conducted in the absence of any commercial or financial relationships that could be construed as a potential conflict of interest.
